# Large-Scale Wearable Sensor Deployment in Parkinson’s Patients: The Parkinson@Home Study Protocol

**DOI:** 10.2196/resprot.5990

**Published:** 2016-08-26

**Authors:** Ana Lígia Silva de Lima, Tim Hahn, Nienke M de Vries, Eli Cohen, Lauren Bataille, Max A Little, Heribert Baldus, Bastiaan R Bloem, Marjan J Faber

**Affiliations:** ^1^ Donders Institute for Brain, Cognition and Behavior Radboud university medical center Nijmegen Netherlands; ^2^ Radboud university medical center Department of Neurology Nijmegen Netherlands; ^3^ CAPES Foundation, Ministry of Education of Brazil Brasília/DF Brazil; ^4^ Intel Advanced Analytics Tel Aviv Israel; ^5^ Michael J Fox Foundation for Parkinson’s Research New York, NY United States; ^6^ Aston University Birmingham United Kingdom; ^7^ Media Lab Massachusetts Institute of Technology Cambridge, MA United States; ^8^ Philips Research Department Personal Health Eindhoven Netherlands; ^9^ Radboud university medical center Radboud Institute for Health Sciences, Scientific Center for Quality of Healthcare Nijmegen Netherlands

**Keywords:** Parkinson’s disease, ambulatory monitoring, signal processing, computer-assisted, wearable sensors

## Abstract

**Background:**

Long-term management of Parkinson’s disease does not reach its full potential because we lack knowledge about individual variations in clinical presentation and disease progression. Continuous and longitudinal assessments in real-life (ie, within the patients’ own home environment) might fill this knowledge gap.

**Objective:**

The primary aim of the Parkinson@Home study is to evaluate the feasibility and compliance of using multiple wearable sensors to collect clinically relevant data. Our second aim is to address the usability of these data for answering clinical research questions. Finally, we aim to build a database for future validation of novel algorithms applied to sensor-derived data from Parkinson’s patients during daily functioning.

**Methods:**

The Parkinson@Home study is a two-phase observational study involving 1000 Parkinson’s patients and 250 physiotherapists. Disease status is assessed using a short version of the Parkinson's Progression Markers Initiative protocol, performed by certified physiotherapists. Additionally, participants will wear a set of sensors (smartwatch, smartphone, and fall detector), and use these together with a customized smartphone app (Fox Insight), 24/7 for 3 months. The sensors embedded within the smartwatch and fall detector may be used to estimate physical activity, tremor, sleep quality, and falls. Medication intake and fall incidents will be measured via patients’ self-reports in the smartphone app. Phase one will address the feasibility of the study protocol. In phase two, mathematicians will distill relevant summary statistics from the raw sensor signals, which will be compared against the clinical outcomes.

**Results:**

Recruitment of 300 participants for phase one was concluded in March, 2016, and the follow-up period will end in June, 2016. Phase two will include the remaining participants, and will commence in September, 2016.

**Conclusions:**

The Parkinson@Home study is expected to generate new insights into the feasibility of integrating self-collected information from wearable sensors into both daily routines and clinical practices for Parkinson’s patients. This study represents an important step towards building a reliable system that translates and integrates real-life information into clinical decisions, with the long-term aim of delivering personalized disease management support.

**ClinicalTrial:**

ClinicalTrials.gov NCT02474329; https://clinicaltrials.gov/ct2/show/NCT02474329 (Archived at http://www.webcitation.org/6joEc5P1v)

## Introduction

Parkinson’s disease (PD) is a progressive and complex neurological disorder. Patients can experience a wide range of motor symptoms and signs, including bradykinesia, tremor, rigidity, and postural instability. Non-motor symptoms include executive dysfunctions, memory disturbances, attention difficulties, and reduced ability to smell [1-3].

The cornerstone of current therapy is based on the replacement of dopamine, but can also include other drugs that play a role in the activation of dopamine receptors [4]. Although these medications initially have good results in disease management, the effects remain successful for a limited period of time. Most patients eventually develop motor complications, such as the wearing-off effect or dyskinesias [5,6]. Some disease symptoms, such as postural instability and voice/speech impairment, are insufficiently (or sometimes not at all) responsive to dopaminergic therapy.

Two major problems hamper the delivery of optimal individual treatment. First, evaluation of day-to-day variations in a complex disease such as PD is difficult when relying solely upon periodic consultations with experts working in a clinical setting [7]. Even when health professionals use specific and validated instruments, such as the Movement Disorder Society - Unified Parkinson's Disease Rating Scale (MDS-UPDRS) [8], the results represent a subjective and episodic *snapshot* taken under well-controlled conditions, which are usually not representative of the patient’s functioning in daily life. More detailed, objective, and reliable knowledge about real-life functioning would greatly improve the quality of individual medical management. Second, virtually all scientific evidence that is presently available to inform PD management stems from biased clinical studies with short follow-up periods in highly selected sub-populations, who were studied under carefully controlled trial conditions [9,10]. As such, this evidence does not reflect the clinical presentation, treatment response, or disease progression in actual daily life.

To overcome these limitations, wearable sensors are emerging as new tools to continuously and longitudinally obtain information from patients in real-life. The accuracy of sensor data for everyday activity recognition (eg, walking, running) in real-life ranges from 58% to 97% [11]. These sensors, typically consisting of embedded accelerometers, have been used successfully to determine PD-related symptoms [12-16]. However, to date these studies have relied upon small sample sizes (n=5 to 43 participants) and short follow-up periods (3 days to 6 months; see Multimedia Appendix 1).

The primary aim of the Parkinson@Home study is to evaluate the feasibility and patient compliance of using wearable sensors to collect data for at least 3 months in a large patient group. A secondary aim of this study is to address the usability of these data for answering clinically relevant research questions (eg, to determine the relationship between sensor-derived measures and clinical measures). Finally, the study aims to build a database for future development and testing of novel algorithms applied to sensor-derived data from PD patients during daily functioning.

## Methods

### Study Design

The Parkinson@Home study is an observational study involving 1000 patients (from whom data will be recorded) and 250 physiotherapists (who will assist in performing the clinical assessments, and who may act as personal coaches during follow-up). Both patients and therapists will be recruited throughout the Netherlands. The study consists of two phases. Phase one aims to assess the feasibility of deploying wearable sensors in a large PD population (n=300). For this purpose, patients will use a number of wearable devices (Pebble smartwatch, Android smartphone, and fall detector) in combination with a customized app (Fox Insight). Follow-up will occur after 3 months (13 weeks), starting from the moment the first data are streamed to the server. In addition to using wearable devices, participants will attend a one-time consultation, during which a detailed clinical assessment will be performed by an experienced physiotherapist or a research team member. This clinical assessment will take place in week 7, or later during the follow-up period. Phase two, which will include an additional 700 participants, aims to collect raw sensor data in order to investigate the usability of these data for answering clinical research questions. This phase will also be used to build a database for future validation of novel algorithms applied to sensor-derived data from PD patients during daily functioning. Patients involved in phase one can also be included in phase two if they wish. Data collection for clinical results and device-based outcomes, as well as the follow-up period, will be identical to phase one. To ensure the success of the raw data collection during phase two, devices and raw data collection strategies used in this phase will be chosen after the evaluation of data collected during phase one.

The study protocol was successfully piloted prior to full study implementation to ensure methodological feasibility. In total 20 Dutch PD patients participated in this pilot, using a set of wearable devices (one smartphone and one smartwatch) and the Fox Insight app. The patients were asked to use these devices for 24 hours, seven days a week, and were followed for four weeks. In total, 58% of patients that were approached agreed to participate. Some patients were reluctant to manage technology and to deal with possible technical problems, which caused them to refrain from participation. All participants (except for two) needed at least one support call for device troubleshooting. Streaming compliance for the sensor data was 88%.

### Inclusion and Exclusion Criteria

#### Patients

The inclusion and exclusion criteria for patients will be kept purposefully broad, in order to represent the full diversity of real-life PD experiences. Inclusion criteria specify that patients must be 30 years of age or older, and be diagnosed with PD by a physician. No exclusion criteria will be applied.

#### Physiotherapists

Physiotherapists who are members of the Dutch ParkinsonNet [17,18] are eligible to participate. ParkinsonNet physiotherapists have received several PD-specific educational training programs, and treat a high number of PD patients each year. Physiotherapists who want to participate should take the official MDS-UPDRS course (provided online by MDS [19], and further in person training provided by the research team) and be able to include and/or assess an average of four PD patients for the study.

### Patient Recruitment Process

We will apply an incremental recruitment strategy. Initially, we will only include patients that already possess a compatible Android/iPhone smartphone. Subsequently, and only if needed, we will include patients that do not possess a smartphone; these patients will be provided with a loaned smartphone device. The reason for this incremental approach is that patients with their own device will likely require less technical support from the research team, as was the case in our pilot study. This strategy will increase the feasibility of complete data collection in a total of 1000 patients.

Patients will be recruited both in the community and through their treating physiotherapists. To reach potential participants in the community, we will use a number of communication channels: (1) the ParkinsonConnect community, an online community for Parkinson’s patients and healthcare professionals involved in their care [18]; (2) the webpage of the Dutch Parkinson Patient Association; (3) an article in the magazine of the Dutch Parkinson Patient Association; (4) presentations about the study to local patient support groups (*Parkinson Cafés*); (5) promotional material for patients will be sent to all ParkinsonNet physiotherapists (approximately 990 individuals), regardless of whether they participate in the study or not, and we will ask them to recruit patients within their practice; and (6) via a study website [20] which provides information about the study. The study website offers both patients and physiotherapists the possibility to sign up for the study online.

After signing up for the study, potential participants will be contacted by phone by a member of the research team, who will provide additional information about the study and check eligibility. If respondents are eligible and willing to participate, they will receive an informed consent form. After the informed consent form has been signed digitally, the research team will provide the participant with all necessary devices and user manuals.

### Recruitment and Training of Physiotherapists

All ParkinsonNet physiotherapists will be contacted by email to inquire about study participation. Should this email not result in adequate numbers of participating physiotherapists, we will personally contact ParkinsonNet physiotherapists by telephone. As with the recruitment process for patients, after signing up via the study website, physiotherapists will be contacted by email or phone to check eligibility.

Once included, physiotherapists must pass the online MDS-UPDRS training successfully [19], as required by the International Parkinson and Movement Disorder Society, which allows them to perform the MDS-UPDRS [8]. After successful completion of the training, physiotherapists will participate in one face-to-face training session, in which they will assess one patient, in order to practice the MDS-UPDRS assessment and consolidate their understanding of the assessment process and study procedures.

### Ethical Aspects and Trial Registration

This study will be conducted in compliance with the Ethical Principles for Medical Research Involving Human Subjects, as defined in the Declaration of Helsinki. The study protocol and communication materials have been approved by the local ethics committee (Commissie Mensgebonden Onderzoek, Arnhem-Nijmegen; NL53034.091.15).

Consent will be obtained by the research team through an innovative online procedure, which includes a compulsory cooling-off period in a digital environment. When the patient is deemed eligible (eg, meets the inclusion criteria specified in the online sign-up form), an information letter and consent form will be send by email. The research team and an independent physician can be approached for questions and verbal explanation. Next, the potential participant has the possibility to confirm participation digitally, via a new URL sent to him/her by email after 48 hours. The URL redirects the patient to the study webpage, where he/she can confirm that they have read all information and that they agree to participate. As recommended by the ethics committee, this final step is blocked for 48 hours after the first email has been sent, to ensure that potential participants take time to consider participation. After the agreement to participate, the participant will see a confirmation message on the study webpage. No signature or scanning of documents will be necessary at this point. The Parkinson@Home study is registered in the ClinicalTrials.gov registry (NCT02474329) [21].

### Wearable Sensors Phase One

#### Pebble Smartwatch

The Pebble is a commercially available smartwatch, with a variety of embedded sensors, such as tri-axial accelerometer, light sensor, and magnetometer. Accelerometers are able to record acceleration along three orthogonal spatial axes, producing acceleration vectors as single data points, and up to 100 acceleration data vectors can be recorded per second. The Pebble smartwatch operating software allows access to the unprocessed *raw* accelerometer data vectors, creating the opportunity for subsequent analyses of this sensor data. In order to obtain continuous accelerometer data, the Fox Insight app will be installed on each smartwatch. The app enables streaming of the accelerometer data to the smartphone, with a sampling frequency of 50 data vectors per second, using the built-in Bluetooth radios of both the smartwatch and smartphone.

#### Fox Insight App

The Fox Insight app is an Android/iPhone app created and developed by Intel Corporation (Tel Aviv, Israel). This app receives 50 accelerometer data points per second from the Pebble smartwatch, and estimates levels of activity, tremor, and sleep movement analyses using dedicated algorithms running within the app. The app presents these estimated quantities to the user by means of graphs and summary reports of the data collected.

**Figure 1:**
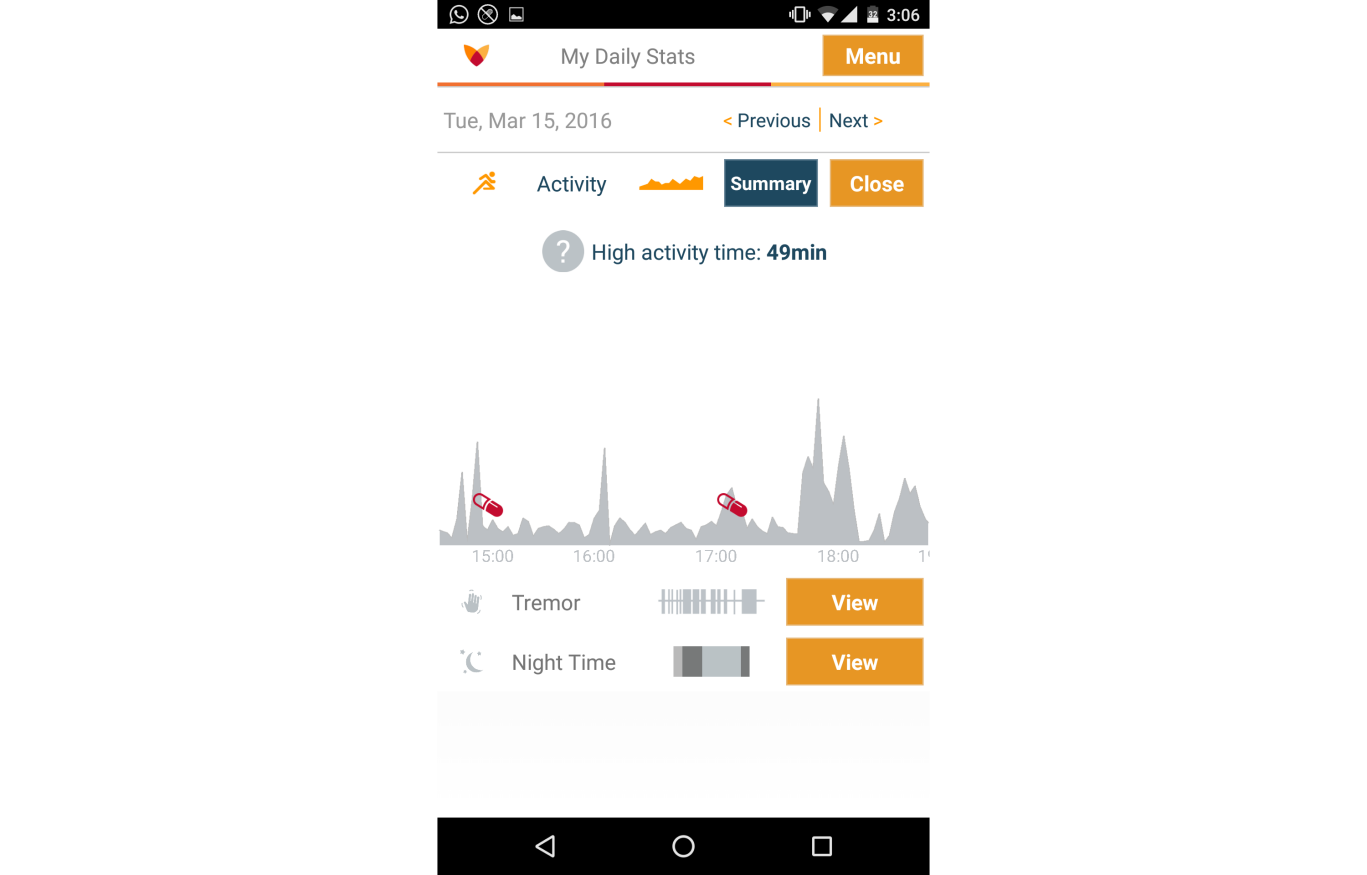
Fox Insight Mobile App activity graph.

**Figure 2:**
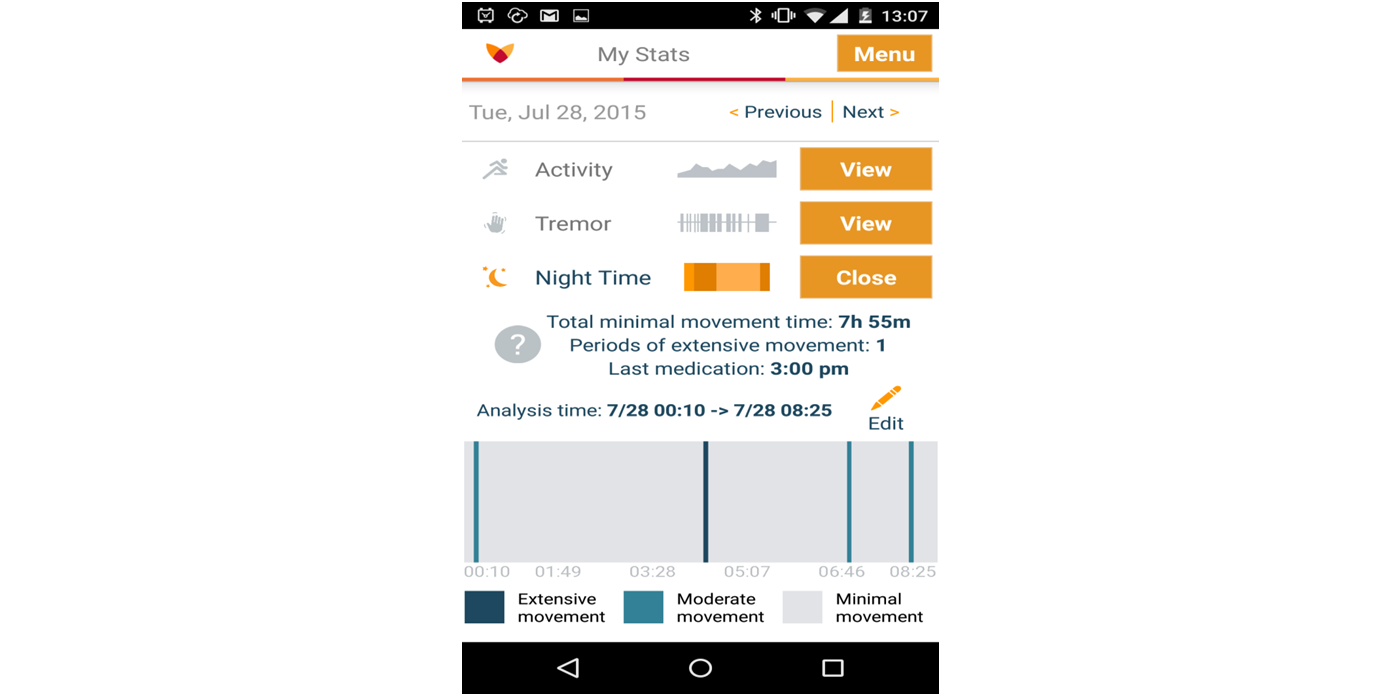
Fox Insight Mobile App sleep analysis graph.

Activity graphs show the level of activity throughout the day (Figure 1). The calculation is performed by aggregations (30 second intervals) of the raw data previously collected. The graph also highlights the moments in time when medication was taken. Daily tremor graphs show how many minutes the patient has experienced tremor during a certain day (a tremor is defined as any movement in the range of 3.5-12Hz). Sleep analysis graphs (Figure 2) show the amount of time that the patient has been active during the sleep time. These graphs provide an impression of the intensity and duration of movements.

**Figure 3:**
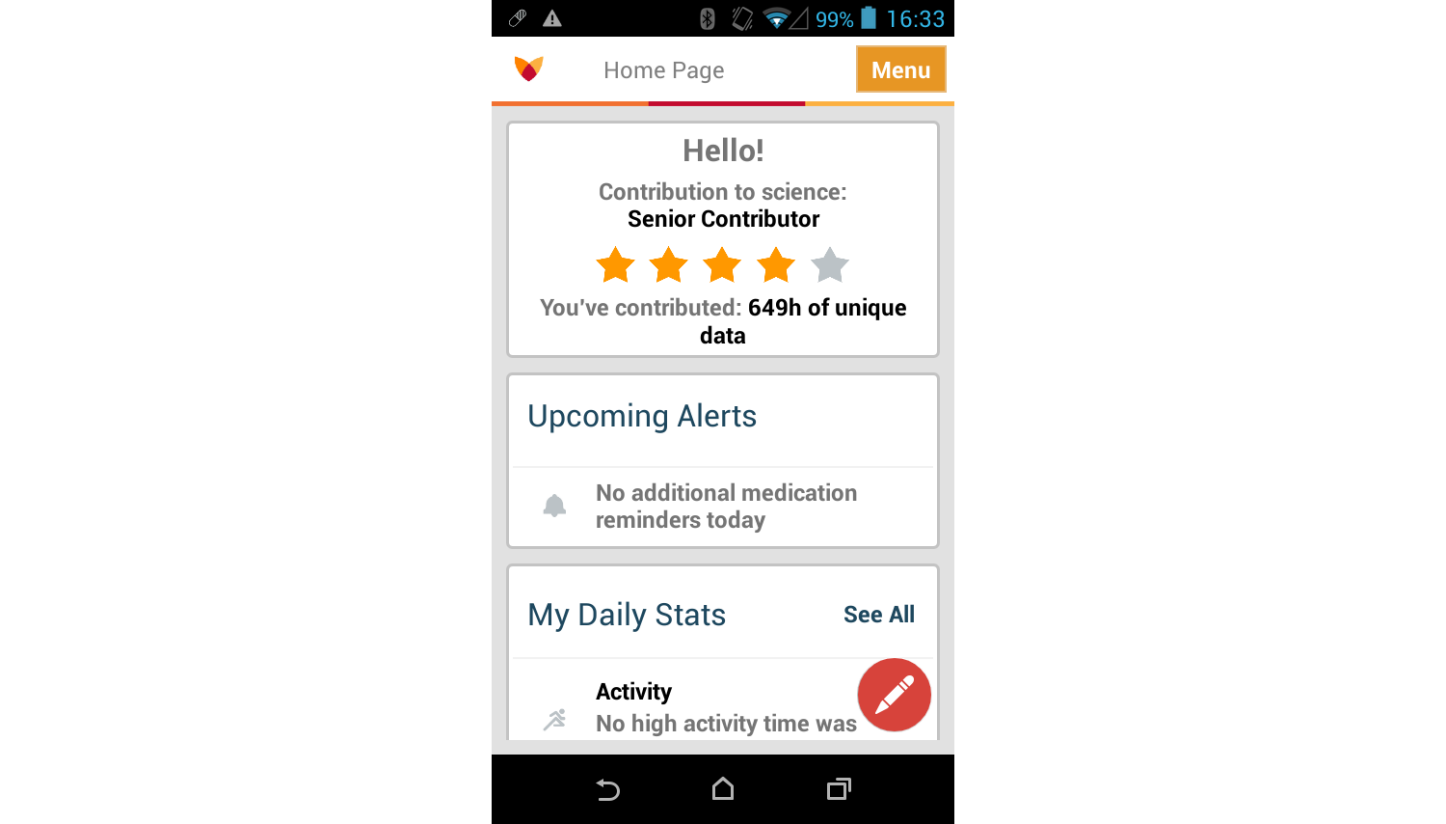
Fox Insight Mobile app main screen.

These estimated quantities are sent every 10 minutes to a cloud-based data platform through an Internet connection on the smartphone. Different mechanisms allow the participants to know whether the data are recorded correctly. First, participants can check the metric graphs (eg, activity graph, sleep analysis, and tremor); these graphs are plotted using the data recorded in the servers, and will only appear if the data were collected. Second, the main app screen (Figure 3) displays how many hours of data the participant has contributed to the study; if this metric does not increase it means the data are not being collected. Finally, participants can view the white *pill* icon in the smartphone task bar; if the icon has a crossing line over it, data are not being actively recorded.

#### Fall Detector

Falls and movement patterns are measured with a pendant device. Patients are given the choice to wear either a fall detector (FD), or the Philips Mobility Monitor (PMM) [22,23]. Both sensors are CE-marked non-medical devices; the PMM is developed by Philips Research. The FD device used in this study is an adapted fall detection device, originally intended for seniors living in their own homes, that was designed to detect falls from stance. The FD device can be worn 24/7, while the PMM is recharged overnight and thus only worn during the day.

The FD device uses multiple sensors and a proprietary analytical algorithm to detect some types of fall events, which are stored in the device. The PMM contains a 3-axial accelerometer and a barometric pressure sensor, with a sampling frequency of 50Hz and 25Hz, respectively. Data are continuously recorded and stored on a micro SD card within the device. Based on these data, information about the daily movements, as well as falls detected, are calculated after read-out at the end of each patient’s trial period.

### Wearable Sensors Phase Two

Devices will be chosen after analysis of the study procedure, and data collection is complete in phase one.

### Technical Support

Patients will have access to extended support, including an installation guide, user manual, and information on the study’s webpage. For the duration of the study, a helpline will be available during working hours to support the installation and device usage, and for troubleshooting.

### Clinical and Feasibility Assessment

Certified physiotherapists will perform the short version of the Parkinson Progression Marker Initiative (PPMI) in order to assess disease status [24]. This assessment includes: the MDS-UPDRS (parts I, III, and IV) for disease rating [8]; the Montreal Cognitive Assessment for cognition [25]; and the Modified Schwab and England Activities of Daily Living Scale for activities of daily living [26].

Additional questionnaires will be completed by the patients: MDS-UPDRS part II for motor experiences of daily living; the Scales for Outcomes in Parkinson’s Disease - Autonomic System (SCOPA-AUT) for autonomic dysfunction [27]; the Geriatric Depression Scale for depressive symptoms [28]; and the Epworth Sleepiness Scale for day sleepiness [29].

To assess feasibility, patients will also complete the System Usability Scale [30] and a satisfaction survey created by the research team, to address patients’ impressions on how well particular features of the app are functioning, and the burden associated with the methodology. An overview of outcomes is provided in Multimedia Appendix 2.

### Data Collection and Management

Due to privacy issues, patients will receive a personal identification code that does not contain any information that relates to the individual. The key-file, connecting personal identification codes to personal information, will be stored on a Radboudumc data server, and only the research team has access to the key-file. The key-file will be stored on a different server from the study data for five years, allowing the research team to contact patients after they have finished the study. We anticipate that our efforts to obtain additional research funding will allow for additional follow-up assessments. The key-file will be destroyed after five years.

Data for the study will be collected in the following ways:

*Data from smartwatch and smartphone*: data will be collected continuously in a coded manner, and will be transferred to Intel’s cloud data storage environment using an Internet connection. The cloud environment is based on Amazon Web Services, and developed and managed by Intel’s Advanced Analytics team. Data from the watch and Fox Insight app will be transferred to the Intel platform using a personal identification code for each patient. Moreover, no personally identifiable data will be entered into the app or sent to this data storage platform.

*Data from the PMM and FD*: data will be collected during the time that patients are not lying in bed. Each FD has a unique identifier, and Philips Research will only receive coded data. No personal information is required to use these devices, and no personal data from patients will be shared with Philips.

*Data from the clinical assessments:* data will be collected by means of paper-based forms, and will be entered manually into an online certified data management system. Forms will only contain personal identification codes.

*Data from support and logistics*: ZenDesk software will be used to support the logistics of the recruitment process, and provide technical support during the follow-up phase. ZenDesk is Internet-based, and data access is authenticated by username and password. All communications with ZenDesk servers use industry-standard Secure Sockets Layer encryption by default, and the ZenDesk servers are located at a different site than the Amazon servers. Therefore, research data is never stored on the same server as patients’ identifying codes.

Patients that complete the clinical assessment and stream data for more than seven days will have their data included in further analyses.

### Data Analyses

#### Phase One

Feasibility and compliance will be addressed using descriptive analyses. For feasibility, the primary outcomes will include the total support time per participant, the number and rate of drop-outs, usability of the system, bias within recruitment strategies, and the type of problems faced by patients. Regarding compliance, the outcome measures include the total hours of sensor data collected per participant, the number of compliant days, and the percentage of time that sensor data were streamed during the follow-up period.

#### Phase Two

The potential for the data to answer clinically relevant research questions will be explored. First, we aim to extract a limited set of outcomes, including: the number, diversity, and performance of physical activities; specific activities (eg, standing, walking, sitting); response fluctuations in relation to drug treatment; and specific motor symptoms (eg, tremor, gait freezing, shuffling, falls).

Additionally, we aim to explore how these outcomes are related to clinical assessments, and to self-monitoring during follow-up (including timing of medication intake and fall incidents). Finally, we aim to extract patterns of disease progression, assess the recognition of disease profiles based on reported symptoms and progression patterns, and address the effect of medication intake on symptoms. In both phases, analyses will be performed using specialized algorithms (when necessary) developed within the Matlab platform, with additional statistical analyses using the R software package.

## Results

### Patient Recruitment Process

Within eight months of recruitment (August, 2015 to March, 2016) the Parkinson@home study received 1164 applications. Among those invited for phase one (n=342), the participation rate was 87.7%, resulting in 300 inclusions. Recruitment strategies through the network of the Dutch Parkinson Association, and a personal approach by the research team or health care providers, have been very successful (Table 1). *Applicants* include all respondents that demonstrated interest in the study, while *participants* include all respondents that were actually included in the study (which excludes dropouts and those who refused to participate). Phase two will begin in September, 2016, and participants will be recruited from the 734 participants placed in the study waiting list.

**Table 1:** Number of applications obtained from each recruitment strategy.

Recruitment Strategy	Applicants n=1164 (%)	Participants n=258 (%)
Online community for patients (ParkinsonConnect)	12 (1.03)	8 (3.10)
Website of the Dutch Parkinson Association	142 (12.19)	20 (7.75)
Article in the magazine of the Dutch Parkinson Association	451 (38.74)	100 (38.75)
Informative presentation at Parkinson Cafés by research team	112 (9.62)	36 (13.95)
Personal invitation by Physiotherapist or Neurologist	198 (17.04)	31 (12.02)
Others	224 (19.24)	51 (19.77)
Not specified	25 (2.14)	12 (4.65)

## Discussion

In this paper we present the rationale and design of the Parkinson@Home study, a large (n=1000) observational cohort study that aims to explore the feasibility and usability of collecting raw sensor data from wearable sensors in patients with PD. There is a pressing need for collection of reliable medical information from PD patients while they perform activities of daily living, due to gaps in knowledge as to why different patients have variable rates of PD progression and different patterns of symptoms [31,32]. It has proven to be extremely difficult to understand such variations, and to capture objective data about the patient’s actual functioning in current clinical practice, which typically consists of episodic and brief clinical evaluations in hospitals.

Gathering data from wearable sensors has high scientific potential and offers several advantages compared to more traditional methods of data collection. Wearable sensors offer the possibility to collect data by self-administered tests, and to objectively monitor PD symptoms and day-to-day variation both remotely and at home [33-35]. The raw sensor data can be analyzed later by specialized algorithms or by algorithms embedded in apps themselves, providing scientific insights for researchers and clinicians. Moreover, data can be collected continuously over a prolonged period of time. For individual PD patients, those data can be used for long-term health monitoring. When applied in a group context, the data may offer a better understanding of PD (eg, by revealing the presence of specific phenotypic subtypes, or by predicting disease progression) [36].

Using wearable sensors also brings about challenges. First, data from sensors are a potential target for invasions of privacy [37]. For example, Global Positioning System-based sensor data can be used to identify the physical location of an individual, and their homes [38]. As a remedy, approaches such as restricting access to the data and anonymizing files have been suggested [39]. To allow for the collection of sensitive data, and to address security issues, the Parkinson@Home project will adopt several precautions, including: coding the data; storing the data on secure servers, separately from personal data; and restricting data use, by only allowing access to authorized researchers within the research team. When making information available to the wider research community, data will be anonymized and access will be granted only through a secure research database. These actions decrease the risk of identification of the patient and inappropriate use of the data.

A second challenge faced in the Parkinson@Home study is the lack of experience that elderly people have with technical devices. This lack of experience affects the acceptance of, and compliance with, the technology [40]. Overcoming this lack of experience in our target population, without introducing a selection bias, will be a challenge. However, we believe that the best approach for this issue is to rely on the willingness of patients to learn and be engaged in the management of their disease, combined with an efficient support model.

In conclusion, this study will generate new insights into the use of wearable sensors in daily living by PD patients, and if the data collection shows potential, it will make a contribution to the integration of self-collected information into clinical practice for PD patients. This study represents the first steps towards building a reliable system that integrates real-life information into clinical decisions.

## Appendix

Multimedia Appendix 1 Overview of studies applying wearable sensors and their use in Parkinson’s disease.

Multimedia Appendix 2 Parkinson@Home study data.

## Abbreviations

FD: fall detector
MDS-UPDRS: Movement Disorder Society - Unified Parkinson's Disease Rating Scale
PD: Parkinson’s disease
PMM: Philips Mobility Monitor
PPMI: Parkinson Progression Marker Initiative
SCOPA-AUT: Scales for Outcomes in Parkinson’s Disease – Autonomic System
