# Supplementary material for: Large-Scale Wearable Sensor Deployment in Parkinson’s Patients: The Parkinson@Home Study Protocol
Source: JMIR Res Protoc. 2016 Aug 26;5(3):e172. doi: 10.2196/resprot.5990 (PMC5018102; doi:10.2196/resprot.5990)
Supplement: Multimedia Appendix 1 [file resprot_v5i3e172_app1.pdf]

Multimedia Appendix 1. Overview of studies applying wearable sensors and their use in Parkinson's disease

Author	Design (1)<br>Environment (2)	Aim (1)<br>Intervention (2)	Type of sensor	N	Follow up period	Conclusions
Arora [2014][40]	1: Cohort<br>2: Home	1: Discriminative validity of self-administered tests for gait and postural sway.<br>2: Gait, posture, voice, reaction time and tapping tests were performed while using a smartphones capable of recording tri-axial acceleration, audio and touch screen tapping events	Smartphone	10 PWP<br>10 healthy controls	1 month	Wearable sensors can provide data that enable to distinguish between healthy subjects and Parkinson's patients with a mean sensitivity of 96.2% (SD 2%) and mean specificity of 96.9% (SD 1.9%).
Personal communication Tsanas, 2012	1: Cohort<br>2: Home	1: Accuracy of speech signals to estimate UPDRS rating.<br>2: Six voice recordings of the sustained phonations 'aaaah', sustained as long as each patient was able to. Voice recordings were captured by using a microphone on a dedicated monitoring device, once a week, for a duration of 6 months.	Mobile phone	42 PWP	6 months	Voice recordings estimate the UPDRS within 3.5 points of the clinicians' assessment.
Patel<br>[2009][13]	1: Cohort<br>2: Laboratory	1: Reliability of using accelerometer data to estimate the severity of symptoms and motor	Accelerometers	12 PWP	Not applicable	Uniaxial accelerometer sensors are able to provide an estimate of clinical scores for tremor, bradykinesia and dyskinesia with an

Author	Design (1)<br><br>Environment (2)	Aim (1)<br><br>Intervention (2)	Type of sensor	N	Follow up period	Conclusions
		complications.<br><br>2: Patients performed the motor assessment part of the UPDRS, in the ON [once] and OFF [6 times] phases, wearing uniaxial accelerometer sensors positioned on the upper and lower limbs.				average estimation error of 3.4%, 2.2%, and 3.2% respectively.
Sharma [2014][12]	1: Cohort<br><br>2: Laboratory	1: To introduce the SPARK wearable system for measuring disease symptoms.<br><br>2: Patients wore a pair of devices (smartwatch and smartphone), and performed tasks to evaluate facial tremors, speech, dyskinesia and freezing of gait. They were also asked to perform tasks included in a digital version of the UPDRS.	Smartphone and smartwatch	5 PWP	Not applicable	The system was able to provide useful features for measuring symptom severity in the real world. Further work in system validation is still in development.
Bachlin<br><br>[2010] [16]	1: Cohort<br><br>2: Laboratory	1: Sensitivity and specificity of detecting freezing of gait events.<br><br>2: Patients wore a set of wearable devices, which are able to detect freezing of gait automatically and provides a cueing sound when this event is detected. The measurement	Accelerometers	10 PWP	Not applicable	Sensitivity of 73% and specificity of 82% in identifying freezing of gait events

Author	Design (1)<br><br>Environment (2)	Aim (1)<br><br>Intervention (2)	Type of sensor	N	Follow up period	Conclusions
		was divided into three parts, and was performed on the same day at the laboratory.				
Griffiths [2012][14]	1: Cohort<br><br>2: Home	1: To test the use of the commercial Kinetigraph algorithm to provide a conventional clinical rating.<br><br>2: Patients wore a device which incorporates a 3-axis accelerometer, for 10 days.	Parkinson's Kinetigraph	34 PWP	10 days	The Kinetigraph algorithm predicted the clinical dyskinesia rating scale with a 95% margin of error of 3.2 units compared with the inter-rater 95% limits of agreement from 3 neurologists of −3.4 to +4.3 units.
Patel [2011][15]	1: Cohort<br><br>2: Laboratory and home	1: To estimate clinical scores for motor symptoms using accelerometers<br><br>2: Subjects performed motor assessments on three days. The first two days of monitoring performed in the clinical setting, third day of monitoring performed in the home setting. Four test sessions are performed on each day of monitoring. During each of these tests, subjects perform a set of tasks from the UPDRS, while wearing a tri-axial accelerometer sensor.	Triaxial accelerometers [SHIMMER ® platform].	5 PWP	3 days with 4 months cooling off period between each day	The sensors and algorithm are able to track longitudinal changes in motor symptoms, by analyzing UPDRS scores using random forest regression, within 0.5 points on a scale of 0-4.

Author	Design (1)<br><br>Environment (2)	Aim (1)<br><br>Intervention (2)	Type of sensor	N	Follow up period	Conclusions
Tsipouras [2012] [42]	1: Cohort<br><br>2: Laboratory	1: Accuracy of an eletronic/automated methodology for measuring levodopa induced dyskinesia.<br><br>2: The methodology is based on the analysis of signals recorded during three major tasks: 1- lying on the bed; 2- rising from the bed and sitting on a chair located near the bed; 3- standing up from the chair and performing a series of tasks.	Accelerometers, gyroscopes and a portable data recorder.	11 PWP<br><br>5 healthy controls	Not applicable	The results obtained indicate that the proposed method is efficient (97.36% classification accuracy) for detecting levodopa induced dyskinesia.
Cancela [2013] [43]	1: Cohort<br><br>2: Laboratory and home	1: To investigate the technical performance of the PERFORM wearable system, a monitoring and health care platform for PD patients.<br><br>2: *Phase 1 and 2*: recordings when wearing the PERFORM system, in a hospital environment.<br><br>*Phase 3*: 2 records of approximately 4 hours wearing the PERFORM system, for five consecutive days, in the patient's home.	Accelerometers and gyroscopes	20 healthy [phase 1]<br><br>36 PWP [phase 2]<br><br>44 PDP and 12 healthy controls [phase 3]	5 days	The PERFORM system, showed an accuracy of 93.73% for the classification of levodopa induced dyskinesia severity, 86% bradykinesia severity, and 87 % for tremor. Regarding usability, 8 out of 24 patients reported extreme discomfort/pain when wearing the system.

Author	Design (1)<br><br>Environment (2)	Aim (1)<br><br>Intervention (2)	Type of sensor	N	Follow up period	Conclusions
Lakshminarayana [2014] [35]	1: Study protocol of NRT<br><br>2: Home	1: To evaluate the impact of using a smartphone and web apps to promote patient self-management as a tool to increase treatment adherence and enhance the quality of clinical consultation.<br><br>2: Group 1: smartphone and internet-enabled PD tracker smartphone app<br><br>Group 2: usual management [treatment] for PDP and their carers.	Smartphone	222 PWP	4 months	Study is currently running.

NRT - Non-randomized trial; UPDRS - Unified Parkinson's Disease Rating Scale; SPARK – Smartphone/Smartwatch system for Parkinson disease; PERFORM - A soPhisticatEd multi-parRametric system FOR the continuous effective assessment and Monitoring of motor status in Parkinson's disease and other neurodegenerative diseases; UPDRS - Unified Parkinson's Disease Rating Scale; PD- Parkinson's Disease; PWP- People with Parkinson's Disease; PERFORM - A soPhisticatEd multi-parRametric system FOR the continuous effective assessment and Monitoring of motor status in Parkinson's disease and other neurodegenerative diseases.
