# Supplementary material for: Large-Scale Wearable Sensor Deployment in Parkinson’s Patients: The Parkinson@Home Study Protocol
Source: JMIR Res Protoc. 2016 Aug 26;5(3):e172. doi: 10.2196/resprot.5990 (PMC5018102; doi:10.2196/resprot.5990)
Supplement: Multimedia Appendix 2 [file resprot_v5i3e172_app2.pdf]

Multimedia Appendix 2. Parkinson@Home study data.

**Study Instrument**	**Outcome measure**	**Type**	**Frequency**
Demographic profile	Age at disease onset	Patient self-report	Once
	Ethnicity	Patient self- report	Once
	Level of education	Patient self- report	Once
	Time since diagnoses	Patient self - report	Once
PPMI: MDS-UPDRS [8]	Non-motor experiences of daily living	Physiotherapist report	Once
	Motor experiences of daily living	Physiotherapist report	Once
	Motor examination	Physiotherapist report	Once
	Motor complications	Physiotherapist report	Once
PPMI: Epworth sleepiness scale [29]	Sleep quality	Patient self-report	Once
PPMI: Geriatric Depression Scale [28]	Depressive behavior	Patient self-report	Once
PPMI: MoCA [25]	Cognitive impairment	Physiotherapist report	Once
PPMI: Schwab and England activities of daily living [26]	Functional level at activities of daily living	Physiotherapist report	Once
PPMI: SCOPA-AUT [27]	Autonomic dysfunctions	Patient self-report	Once
Fox Insight app	Medication intake (compliance)	Patient self-report	Multiple time points
	Falls	Patient self-report	Multiple time points
	Percentage of sensor data streaming in 3 months (compliance)	Processed Accelerometer	Multiple time points
Smartwatch (Pebble)	Time that the patient is active during the day	Processed Accelerometer	0.2Hz
	Level of physical activity during the day	Processed Accelerometer	0.2 Hz

Study Instrument	Outcome measure	Type	Frequency
	Hours during the day where the patient had tremor	Processed Accelerometer	0.03 Hz
	Amount of movements during sleep time	Processed Accelerometer	0.003 Hz
	Raw sensor data	Raw accelerometer	50 Hz
PMM/FD	Number of fall events	Processed Accelerometer	Depending on the number of detected fall events
Zendesk	Time spent providing troubleshooting telephone support per patient (feasibility)	Study support team indicators	Multiple time points
	Inclusion rate (feasibility)	Study support team indicators	Once
	Drop-out rate (feasibility)	Study support team indicators	Once
	Type of technical and/or study procedure problem encountered by patients (feasibility)	Patient self-report	N/A
	Patients' socioeconomic status (SES; possible biases in the population)	Patient self-report	Once
System Usability Scale [28]	Usability and learnability of the Fox Insight app	Patient self-report	Once
Satisfaction survey	User's satisfaction with the system (smartwatch and app)	Patient self-report	Once
	Patients' sociodemographic details (e.g. gender, disease duration, age and previous	Patient self-report	Once

Study Instrument	Outcome measure	Type	Frequency
	experience with smartphones; possible biases in the population)		

PPMI - Parkinson Progression Marker Initiative; MDS-UPDRS - Movement Disorder Society - Unified Parkinson's Disease Rating Scale; MoCA - Montreal Cognitive Assessment for cognition; SCOPA-AUT- Scales for Outcomes in Parkinson’s Disease – Autonomic System for autonomic dysfunction
